# The effect of stimulation therapy and donepezil on cognitive function in Alzheimer’s disease. A community based RCT with a two-by-two factorial design

**DOI:** 10.1186/1471-2377-12-59

**Published:** 2012-07-19

**Authors:** Fred Andersen, Matti Viitanen, Dag S Halvorsen, Bjørn Straume, Tom Wilsgaard, Torgeir A Engstad

**Affiliations:** 1 Department of Community Medicine, University of Tromsø, (Breivika), 9037 and Árran Lulesami Centre, (Sentrum), Drag, 8270, Tromsø, N-8270, Norway; 2 Department of Geriatrics, Karolinska Institutet, (Huddinge), Stockholm, (141 86) Sweden, and University of Turku, (Municipality hospital), Turku, 20520, Finland; 3 Department of Medicine, University hospital, (Breivika), Tromsø, 9038, Norway; 4 Department of Community Medicine, University of Tromsø, (Breivika), Tromsø, 9037, Norway; 5 Department of Geriatrics, University Hospital, (Breivika), Tromsø, 9038, Norway

**Keywords:** Alzheimer’s disease, Symptomatic treatment, Postponement of cognitive deterioration

## Abstract

**Background:**

Progressive neurodegeneration in Alzheimer’s disease (AD) induces cognitive deterioration, and there is controversy regarding the optimal treatment strategy in early AD. Stimulation therapy, including physical exercise and cholinesterase inhibitors are both reported to postpone cognitive deterioration in separate studies. We aimed to study the effect of stimulation therapy and the additional effect of donepezil on cognitive function in early AD.

**Method:**

Design: A two-by-two factorial trial comprising stimulation therapy for one year compared to standard care to which a randomized double-blinded placebo controlled trial with donepezil was added.

Setting: Nine rural municipalities in Northern Norway.

Participants: 187 participants 65 years and older with a recent diagnosis of mild or moderate AD were included in the study of which 146 completed a one-year follow-up. INTERVENTIONS: In five municipalities the participants received stimulation therapy whereas participants in four received standard care. All participants were randomised double-blindly to donepezil or placebo and tested with three different cognitive tests four times during the one-year study period.

Main outcome: Changes in MMSE sum score.

Secondary outcome: Changes in ADAS-Cog and Clock Drawing Test.

**Results:**

MMSE scores remained unchanged amongst AD participants receiving stimulation therapy and those receiving standard care. The results were consistent for ADAS-Cog and Clock Drawing Test. No time trend differences were found during one-year follow-up between groups receiving stimulation therapy versus standard care or between donepezil versus placebo.

**Conclusion:**

In rural AD patients non-pharmacological and pharmacological therapy did not improve outcome compared with standard care but all groups retained cognitive function during one year follow-up. Other studies are needed to confirm these results.

**Trial registration:**

ClinicalTrials.gov (Identifier: NCT00443014). EudraCT database (no 2004-002613-37).

## Background

Alzheimer’s disease (AD) is a progressive neurodegenerative disorder causing cognitive impairment in millions of elderly worldwide. Clinical practice today includes symptomatic treatment with stimulation therapy and/or pharmaceutical intervention with cholinesterase inhibitors (ChEI) due to lack of causal treatment [1,2].

Non-pharmaceutical interventions on AD such as exercise training, occupational therapies and cognitive stimulation have been examined in studies of various design, size and duration. Until recently such interventions have not been tested in large-scale studies [3]. A Cochrane review (2003) described limited effects of cognitive stimulation therapy alone[4], but a meta-analysis including 30 trials studying the effect of exercise training in AD patients showed a significant effect on cognitive and functional performance as compared to the control group [5]. Graff and colleagues showed that occupational therapy improved cognitive function significantly and reduced the burden on caregivers [6]. Despite controversies, ChEIs have been promoted during the last fifteen years as symptomatic treatment of mild to moderate AD. Several meta-analyses have reported a modest effect of ChEI on cognition [7-9]. A head to head comparison of the effect of ChEI versus stimulation therapy has to our knowledge not been performed [3] although the combined effect of donepezil and stimulation therapy compared to control groups is examined in a few studies [10-12].

The main purpose of this study was to examine the effect of stimulation therapy on cognitive function in community dwellers with mild to moderate AD in Northern Norway. A secondary aim was to examine whether donepezil increased the effect of stimulation therapy on cognition.

## Method

### Design

The present study has a double design; - an open intervention with stimulation therapy to which a randomised double blinded and placebo-controlled clinical trial (RCT) with donepezil is added, constituting a two-by-two factorial design.

### Participants

General practitioners (GPs) recruited 87 and population-based screening 100 participants to the study, all with a recent diagnosis of AD at inclusion. 45 of these 187 participants (24%) were nursing home residents served by the primary health care in the participating municipalities and 142 lived in their own homes. 146 participants accomplished a one-year follow-up. At baseline no significant differences between subgroups were found with respect to age, gender, cognitive function, neuropsychiatric symptoms, activities of daily living, drug consumption, number of co-morbidities or education level (Table 1 and Table 2).

**Table 1:** Baseline cognitive, neuropsychological and ADL function according to follow-up groups

**Groups**	**n**	MMSE ± SD*	ADAS-cog ± SD*	CDT** ± SD*	BI§ ± SD*	NPI‡ ± SD*
Municipality						
Stimulation	103	22.9 ± 4.6	18.9 ± 8.7	2.85 ± 1.2	6.08 ± 9·6	18.6 ± 2.6
Standard care	77	23.5 ± 4.3	17.1 ± 7.0	2.84 ± 1.2	8.48 ± 10·5	18.6 ± 3.2
p-value		0.34	0.22	0.96	0.12	0.995
Drug						
Donepezil	90	23.2 ± 4.2	18.6 ± 7.7	2.86 ± 1.1	7.48 ± 11·4	18.9 ± 2.1
Placebo	90	23.1 ± 4.8	17.9 ± 8.4	2.84 ± 1.2	6.70 ± 8·9	18.4 ± 3.4
p-value		0.83	0.55	0.95	0.61	0.29
Combination						
Donepezil	53	22.9 ± 4.5	19.2 ± 8.7	2.92 ± 1.1	6.29 ± 11·3	18.7 ± 2.3
+ stimulation						
Placebo						
+ Standard care	40	23.3 ± 4.9	17.2 ± 8.0	2.93 ± 1.2	7.76 ± 10·4	18.1 ± 4.0
p-value		0.64	0.24	0.998	0.53	0.45
Head to head						
Stimulation therapy	50	22.9 ± 4.7	18.5 ± 8.7	2.78 ± 1.3	5.85 ± 7·4	18.6 ± 2.8
Donepezil	37	23.7 ± 3.7	17.6 ± 5.8	2.76 ± 1.3	9.29 ± 10·8	19.1 ± 1.8
p-value		0.36	0.62	0.93	0.09	0.31

*Mean values ± Standard deviation. **CDT = Clock Drawing Test scale 0–4 (4 best function) §BI = Barthel Index, scale 0–20 (20 best function). ‡ NPI = Neuropsychiatric Inventory, Scale 0–144 (144 worst case).

**Table 2:** Baseline demographic characteristics according to follow-up groups

**Groups**	Age ± SD*	**Gender**	**Education**	**Living**		
		**Female**	**≤ 7 yerars**	**Couple**	**Comorbidity**	**Drug use**
		**n (%)**	**n (%)**	**n (%)**	± SD*	± SD*
Municipality						
Stimulation	81.6 ± 6.7	59(57)	88(85)	48(46)	1.82 ± 1.7	4.89 ± 3.3
Standard care	4.89 ± 3.3	54(65)	65(79)	37(45)	1.65 ± 1.1	5.40 ± 3.9
p-value	0.12	0.25	0.34	0.83	0.45	0.34
Drug						
Donepezil	80.80 ± 6.8	62(67)	82(89)	39(42)	1.90 ± 1.7	5.10 ± 3.3
Placebo	80.85 ± 7.3	50(54)	70(75)	46(49)	1.59 ± 1.2	5.16 ± 3.8
p-value	0.96	0.07	0.014	0.30	0.15	0.90
Combination						
Donepezil + stimulation	81.32 ± 7.0	31(60)	47(90)	28(54)	2.02 ± 2.0	4.92 ± 2.8
Placebo + Standard care	79.81 ± 8.1	22(54)	29(71)	19(46)	1.51 ± 1.1	5.41 ± 3.9
p-value	0.34	0.57	0.015	0.47	0.14	0.48
Head to head						
Stimulation therapy	81.89 ± 8.3	27(53)	40(78)	24(47)	1.61 ± 1.4	4.88 ± 3.7
Donepezil	80.17 ± 6.4	28(74)	32(86)	15(39)	1.76 ± 1.3	5.26 ± 4.0
p-value	0.21	0.046	0.33	0.45	0.61	0.65

*Mean values ± standard deviation.

The recruitment methods and demographic characteristics are described in detail in a previous paper [13]. Inclusion criteria were individuals aged 65–100 years with a recent diagnosis of AD and Mini-Mental-State-Examination (MMSE) [14] sum score at least 10 points. A standard MMSE protocol translated into Norwegian was used and the protocol was not changed during the study period. At entry 43 participants tested between 10 and 20 MMSE points, 92 participants tested between 21 and 25 points and 52 participants tested 26 MMSE points or more.

**Figure 1:**
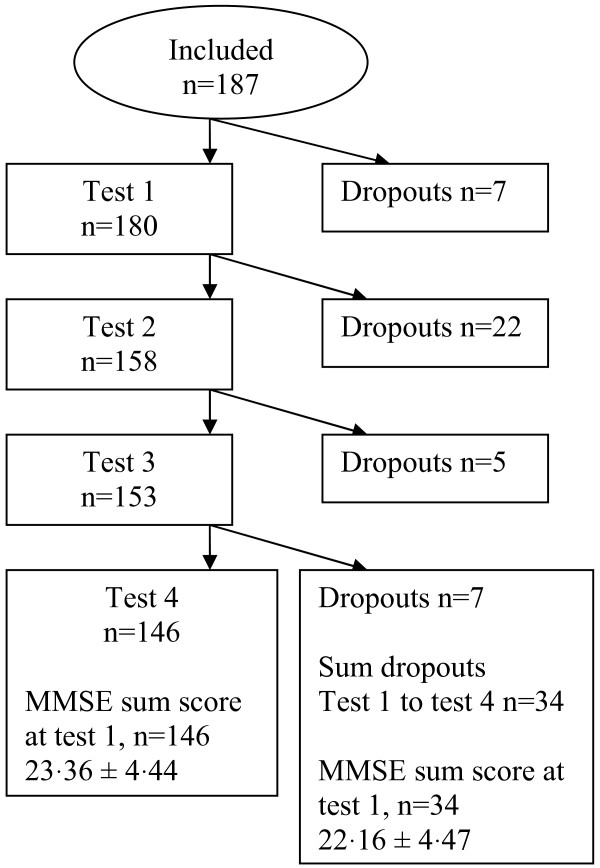
Flowchart of dropouts during follow-up.

Exclusion criteria were dementia other than AD, previous use of donepezil, behavioural disturbances making co-operation and cognitive testing impossible, inability to understand the purpose of the study, any expressed reluctance to participate, or relatives or caregivers disapproving participation. The study period lasted for 39 months of which 27 involved recruitment. All together 41 participants (22%) dropped out, seven prior to the first test and 34 between the first and the fourth (Figure 1).

The study was community based and run on municipality level. Nine rural municipalities in Northern Norway with 70000 inhabitants were engaged. The age group ≥65 years in these communities constituted 11807 individuals. The municipalities were divided into two groups. Participants from five municipalities received stimulation therapy whereas the participants in the remaining four received standard care. The municipalities were selected to the study and allocated to stimulation therapy or standard care according to criteria such as number of inhabitants, age distribution and ethnical homogeneity. Professional competence level and a primary health care organized in accordance with the principles of good clinical practice in each participating municipality were required. Choosing maximum distance and pursuing the least contact possible between municipalities offering stimulation therapy or standard care intended to minimize the risk of dilution. These selection criteria were difficult to apply by random allocation, and each municipality was assigned to the intervention or control municipality group based on study staff consensus.

### Randomisation and masking

**Figure 2:**
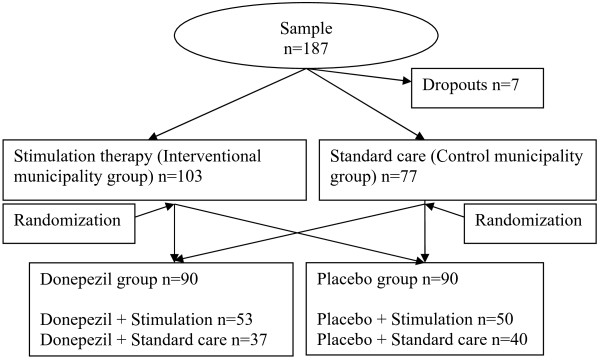
Flowchart of the study design and sample size divided on subgroups.

The Clinical Research Centre at the University Hospital in Tromsø allocated all participants to donepezil or placebo (drug groups) in a randomised manner, in blocks of 4 to 6, by (Figure 2). The treatment assignments were blinded to all study personnel and participants.

### Diagnosing Alzheimer’s disease

A standardised testing program according to national guidelines was used. A semi-structured interview of the participants concentrating on the onset and the course of memory impairment, visuo-spatial disturbances, speech difficulties, executive dysfunctions, and problems with activities of daily living was performed. A family member or a caregiver completed or extended the medical history by adressing the Informant Questionnaire-Cognitive Decline in the Elderly (IQ-CODE) [15] which also assess social consequences of the cognitive impairment. Cognitive function was tested with MMSE and Clock Drawing Test (CDT) [16]. Depressive symptoms were assessed with a semi-structured questionnaire in addition to Montgomery and Aasberg Depression Rating Scale (MADRS) [17] testing AD participants with a MMSE sum score exceeding 21 points. A neurological examination, blood tests and cerebral computed tomography (CT) were performed. The diagnostic procedures for participants recruited by screening were similar to those used in general practice.

GPs and geriatric specialists diagnosed dementia and Alzheimer’s disease according to the ICD-10 criteria [18] and the Statistical Manual of Mental Disorders fourth edition (DSM-IV-TR). Diagnostic discrepancies were discussed with a geriatric colleague both advised by National Institute of Neurological and Communicative Disorders and Stroke-Alzheimer Disease and Related Disorders (NINCDS-ADRDA) criteria [19] for probable AD and solved by consensus. A third specialist (MV) was consulted if disagreement continued.

### Outcome measures

The primary outcome was changes in MMSE sum score. In order to compare with other studies the cognitive tests were supplemented with Alzheimer’s Disease Assessment Scale, Cognitive (ADAS-Cog) [20]. A change in ADAS-Cog and CDT were secondary outcomes. Basic activities of daily living were assessed with Barthel Index (BI) [21]. NeuroPsychiatric Inventory (NPI) [22] was used to identify psychiatric symptoms.

During one-year follow-up MMSE, CDT and ADAS-Cog were performed at baseline and at 4, 8 and 12 months, and all other tests at baseline and at 12 months. Two test technicians performed all testing after supervision and training at The Department of Geriatrics, University Hospital in Northern Norway. To improve intra and inter-rater reliability they observed and evaluated each other by testing a number of patients with MMSE, ADAS-Cog and NPI prior to the study onset.

### Intervention

#### Stimulation therapy

A program of stimulation therapy including physical activities, cognitive, sensory and social stimulation was developed and adjusted to each participant taking cognitive and physical function, educational level and professional background into consideration. The program included systematically performed and intensified daily activities like walking, domestic work, regular reading of books and papers, training in fitness rooms, dancing, crossword puzzles, listening to music, and regular participation in the social life of the community. A few more sophisticated activities such as reminiscence groups, Sudoku, aroma therapy and sensory garden were added, between which the participants could move freely. This stimulation therapy was carried out for a minimum of 30 minutes 5 days a week for one year (maximum 250 sessions a year) in close co-operation with the participant and his/her family or trained health providers. Each session of stimulation therapy was described in a log which was submitted to the study site weekly. These were then compared to the pre-plan individual stimulation program and assessed consecutively, approved or rejected by the study staff. In nursing homes the employee conducted the stimulation therapy while community nurses or other caregivers (family members or even neighbors) guided by the nurses were responsible for the stimulation therapy of community dwellers not regularly receiving community health care. The stimulation program was monitored and adjusted according to functional abilities and interests of the participants during the period of intervention. Nursing home residents and community dwellers received the same stimulation program with an exception for the individual adjustment. Participants living in municipalities allocated to standard care did not receive any organized stimulation.

#### Drug

Having received the randomization codes the pharmacy at the hospital in Bodø distributed the drugs to the patient or the caregiver according to a prescription from the family physician. All participants were prescribed donepezil or placebo once daily. Passing four weeks the dosage was increased from 5 to 10 mg. Adverse events were recorded consecutively.

### Ethics and approvals

The present study was approved by the national authorities including The Regional Committee for Medical Research Ethics in Northern Norway, The Privacy Ombudsman for Research, The Directory of Health and Social Welfare and The Norwegian Medicine Agency with registration in the EudraCT database (no 2004-002613-37). Each participant gave a written informed consent co-signed by a spouse, a close relative or a guardian. The national authorities listed above approved the consent formula and the study was registered as an International Standard Randomized Controlled Trial within ClinicalTrials.gov (Identifier: NCT00443014). In October 2008 The Norwegian Medicine Agency conducted an inspection according to the principles of Good Clinical Practice in a randomized clinical trial. All remarks from this assessment, including monitoring routines were closed and approved.

All the publications from this study comply with The CONSORT statements 2010 and the updated Uniform Requirements for Manuscripts Submitted to Biomedical Journals.

### Statistics

Statistical analyses were carried out using SPSS version 15.0 and 18 (SPSS Inc. Chicago, US). Differences in demographic characteristics between municipality and medicine groups were assessed by independent sample t-tests or Chi-square. Repeated measures analyses assessed differences in time-trends between groups of participants completing one-year follow-up. Linear mixed models were used to assess time-trends in cognitive function over four time points and to assess differences in time-trends between groups of participants with and without baseline cognitive function as covariate. Including an unstructured covariance matrix to the model controlled for possible dependences between repeated observations. In the municipality groups, time trend differences in cognitive function were assessed between the stimulation group and controls, and differences in the medicine groups were assessed between donepezil treatment group and placebo. Finally, the subgroup treated by stimulation therapy and donepezil was compared to the subgroup receiving usual care and placebo. Model assumptions were assessed by means of residual analyses. The statistical analyses were performed according to intention to treat, per protocol and subgroup analyses in order to estimate homogeneity and consistency of the data. In sensitivity analyses we included municipality as random effect in the linear mixed models in order to control for possible clustering of data within the municipalities.

## Results

No significant time trend differences in cognitive performance were observed between participants receiving stimulation therapy or standard care during one year, assessed by MMSE (primary outcome), CDT and ADAS-Cog (secondary outcome). The cognitive test performances remained unchanged during the study period, and addition of donepezil or placebo to the study groups did not alter the results (Table 3 and Table 4). Repeated measures analyses were consistent with the linear mixed model analyses (Data not shown).

**Table 3:** Mean cognitive function in the municipality and drug groups by follow-up time point. Intention to treat analyses

**Groups**	**Moments of follow-up**					
	**Baseline, n = 180**	**4 months, n = 158**	**8 months n = 153**	12 months,* n = 146	**p-value**^**1**^	**p-value**^**2**^
MMSE ± SD**						
Municipality						
Stimulation (n = 103)	22.9 ± 4.6	22.1 ± 5.0	22.9 ± 4.5	22.6 ± 5.2	0.15	0.017
Standard care (n = 77)	23.5 ± 4.3	23.8 ± 4.	24.4 ± 4.0	23.9 ± 4.3		
Drug						
Donepezil (n = 90)	23.2 ± 4.2	23.3 ± 4.5	23.8 ± 4.3	23.0 ± 4.9	0.31	0.21
Placebo n = 90	23.1 ± 4.1	22.9 ± 4.8	23.4 ± 4.3	23.3 ± 4.8		
Clock Drawing Test ± SD**						
Municipality						
Stimulation	2.9 ± 1.2	2.9 ± 1.1	2.8 ± 1.1	3.0 ± 1.1	0.21	0.071
Standard care	2.8 ± 1.2	3.0 ± 1.2	3.1 ± 1.1	3.1 ± 1.1		
Drug						
Donepezil	2.9 ± 1.2	2.8 ± 1.2	2.8 ± 1.2	3.0 ± 1.0	0.56	0.26
Placebo	2.8 ± 1.1	3.0 ± 1.1	3.0 ± 1.0	3.1 ± 1.1		
ADAS-cog ± SD**						
Municipality						
Stimulation	18.9 ± 8.7	18.5 ± 8.4	17.0 ± 9.1	16.8 ± 8.5	0.23	0.11
Standard care	17.4 ± 7.0	16.1 ± 6.9	15.7 ± 8.2	15.9 ± 8.0		
Drug						
Donepezil	18.6 ± 7.6	17.7 ± 7.8	16.3 ± 8.8	16.3 ± 8.4	0.59	0.24
Placebo	17.9 ± 8.4	17.1 ± 7.9	16.5 ± 8.6	16.4 ± 8.2		

*n varies due to dropouts during follow-up. ** Standard deviation. p-value^1^ = Equal time trends between groups. p-value^2^ = Equal time trend between groups adjusted for baseline cognitive function.

**Table 4:** Estimated change in cognitive function by follow-up time point and according to drug groups and municipality groups*

	**Estimated change (95% CI) versus baseline at**			**p-values**	
**Groups**	**4 months**	**8 months,**	**12 months**	**P**^**1**^	**P**^**2**^
MMSE					
Drug groups					
Placebo	−0.402 (−0.988, 0.185)	−0.015 (−0.584, 0.553)	−0.335 (−1.046, 0.375)	0.208	0.284
Donepezil	0.145 (−0.440, 0.730)	0.411 (−0.166, 0.988)	−0.470 (−1.181, 0.241)		
Municipality groups					
Standard care	0.198 (−0.429, 0.826)	0.790 ( 0.190, 1.389)	0.156 (−0.588, 0.901)	0.017	0.508
Stimulation	−0.380 (−0.933, 0.172)	−0.272 (−0.807, 0.262)	−0.841 (−1.510, -0.172)		
Clock Drawing Test					
Drug groups					
Placebo	0.103 (−0.081, 0.288)	0.124 (−0.082, 0.330)	0.134 (−0.079, 0.347)	0.264	0.570
Donepezil	−0.016 (−0.200, 0.169)	−0.101 (−0.309, 0.107)	0.031 (−0.183, 0.245)		
Municipality groups					
Standard care	0.120 (−0.076, 0.317)	0.210 (−0.008, 0.428)	0.234 ( 0.011, 0.456)	0.071	0.257
Stimulation	−0.017 (−0.191, 0.158)	−0.144 (−0.339, 0.050)	−0.038 (−0.242, 0.165)		
ADAS-cog					
Drug groups					
Placebo	−0.383 (−1.412, 0.646)	−1.126 (−2.201, -0.051)	−0.492 (−1.635, 0.650)	0.235	0.886
Donepezil	−1.027 (−2.053, -0.001)	−1.948 (−3.033, -0.862)	−1.510 (−2.661, -0.359)		
Municipality groups					
Standard care	−1.515 (−2.597, -0.433)	−1.736 (−2.889, -0.582)	−1.619 (−2.830, -0.407)	0.108	0.242
Stimulation	−0.069 (−1.030, 0.893)	−1.380 (−2.407, -0.352)	−0.493 (−1.590, 0.603)		

* Linear mixed models adjusted for baseline cognitive score.

P^1^, p-value for overall equality of change between the groups.

P^2^, p-value for test of interaction between group and time, i.e. test for constant differences between groups over time.

When comparing stimulation therapy and donepezil head to head, time-trend analysis showed non-significant changes for CDT and ADAS-Cog, whereas the MMSE test showed borderline significance (p = 0.042) (Table 5). A subgroup analysis comparing the extremes, the combined effect of stimulation therapy and donepezil versus standard care and placebo, did not reveal any significant time-trend differences in cognitive performance (Table 6). Including municipality as a random factor did not change the main result of the study (data not shown).

**Table 5:** Mean cognitive function by follow-up time point and according to a head to head comparison between donepezil versus stimulation therapy

**Groups**	**Time points**				**p-value**^**1**^	**p-value**^**2**^
	**Baseline n = 87**	**4 months n = 76**	**8 months n = 74**	12 months* n = 69		
	MMSE ± SD**					
Stimulation therapy	22.9 ± 4.5	22.2 ± 5.1	23.0 ± 4.0	22.7 ± 4.8	0.040	0.0016
Donepezil	23.7 ± 3.7	23.8 ± 3.8	25.1 ± 3.0	23.6 ± 3.8		
	Clock Drawing Test ± SD**					
Stimulation therapy	2.8 ± 1.3	2.9 ± 1.2	2.8 ± 1.0	2.9 ± 1.1	0.940	0.713
Donepezil	2.8 ± 1.3	2.7 ± 1.3	2.8 ± 1.2	2.9 ± 1.1		
	ADAS-cog ± SD**					
Stimulation therapy	18.5 ± 8.7	17.9 ± 8.6	16.7 ± 8.8	17.6 ± 8.6	0.225	0.060
Donepezil	17.6 ± 5.8	15.8 ± 6.9	15.1 ± 7.9	16.7 ± 8.1		

n varies due to missing values ** Standard deviation. p-value^1^ = Equal time trends between groups. p-value^2^ = Equal time trend between groups adjusted for baseline cognitive function.

**Table 6:** Mean cognitive function by follow-up time point and according to donepezil added to stimulation therapy versus placebo added to standard care

**Groups**	**Time points**					
	**Baseline n = 87**	**4 months n = 76**	**8 months n = 74**	12 months* n = 69	**p-value**^**1**^	**p-value**^**2**^
MMSE ± SD**						
Donepezil + stimulation therapy	22.9 ± 4.5	22.9 ± 4.5	22.9 ± 4.9	22.5 ± 5.5	0.443	0.449
Placebo + standard care	23.3 ± 4.9	23.8 ± 4.2	23.6 ± 4.6	24.1 ± 4.7		
Clock Drawing Test ± SD**						
Donepezil + stimulation therapy	2.9 ± 1.1	2.9 ± 1.1	2.8 ± 1.2	3.1 ± 1.0	0.064	0.035
Placebo + standard care	2.9 ± 1.2	3.2 ± 1.0	3.3 ± 1.0	3.3 ± 1.1		
ADAS-cog ± SD**						
Donepezil + stimulation therapy	19.3 ± 8.7	19.2 ± 8.2	17.2 ± 9.5	16.1 ± 8.7	0.554	0.458
Placebo + standard care	17.2 ± 8.0	16.3 ± 7.1	16.3 ± 8.5	15.1 ± 7.9		

* n varies due to missing values ** Standard deviation. p-value^1^ = Equal time trends between groups. p-value^2^ = Equal time trend between groups adjusted for baseline cognitive function.

Baseline demographic characteristics were well balanced between compared groups with respect to, cognitive performance, activities of daily living (ADL), neuropsychiatric symptoms (NPI) (Table 1), age, gender, social living, drug consumption and co-morbidities (Table 2). The educational level was significantly lower in the donepezil group compared to placebo (Table 2). Of 187 study participants, 146 completed one year follow-up, and 41 withdrew due to disease progression (n = 8), co-morbidity (n = 8), death (n = 7), or unknown reason (n = 18) (Figure 1). The dropouts were equally distributed between subgroups. At entry the dropouts were older (82.5 ± 7.1 versus 80.4 ± 6.9 years), and more cognitively impaired (MMSE 21.17 ± 4.1 versus 23.48 ± 3.7) compared to those completing the study period.

22 participants (25%) dropped out from the donepezil group due to adverse reactions compared to 8 (10%) in the placebo group, p = 0.008 (data not shown). 17 participants in the donepezil group reported gastrointestinal reactions, especially anorexia, diarrhoea and nausea compared to 6 in the placebo group. Participants using donepezil reported depression, dizziness, nightmare and headache whereas these symptoms were uncommon in the placebo group. In two cases the adverse reactions were temporarily and the medication could be resumed. In the other cases the symptoms remained and the drug treatment had to be interrupted.

## Discussion

In this one-year trial, there were no significant changes in cognitive performance between AD participants receiving stimulation therapy or standard care. To our surprise, both groups retained their cognitive function and the results were consistent for three different cognitive tests as assessed at quarterly sessions during the study period. Among the participants receiving standard care, we expected an annual decline of 2–3 MMSE points, or an increase of 5–12 ADAS-Cog points, which is the natural course of AD [23,24].

Our results are in agreement with others [25,26] who reported small changes in mean MMSE score in controls receiving standard care. This is in contrast with Requena et al [10], who reported a significant decline in mean MMSE score from 19.4 (SD 4.9) to 13.1 (SD 5.9) after one year without intervention, but an increase in mean MMSE score from 19.4 (SD 8.2) to 21.9 (SD 7.9) after one year for AD subjects receiving stimulation therapy. However, these studies are limited by short follow up [25], retrospective design, poorly defined controls [26], small sample size and an open design [10].

In our study, there were no differences in cognitive performance between the groups given donepezil or placebo, irrespective of stimulation therapy or standard care. Chapman et al [12] and Matsuda et al [11] reported non-significant changes in mean MMSE score in the combined treatment group given donepezil and stimulation therapy after one year (−1.3 and +0.3 respectively), but a significant reduction in cognitive performance was observed in the donepezil only group (−2.9 and −2.0 respectively). Requena et al reported improved mean MMSE score (+1.5 points) after one year and a minimal reduction after two years in the combined treatment group (−1.3 points), whereas AD participants receiving donepezil without specific stimulation and AD participants in the control group had significantly lower MMSE score (−3.4 and −6.3, respectively). Our results partially support these studies [10-12] as the effect of stimulation on cognitive performance, with or without donepezil, did not deteriorate after one year. However, unlike Requena et al, our participants receiving donepezil or placebo added to standard care retained cognitive performance after one year. The differences could be due to smaller sample size and greater variation in cognitive function among participants in the Requena et al study compared to the present study. Differences in baseline cognitive function between mild and moderate AD may also give different cognitive deterioration slopes.

In a recent systematic review, Olazarán et al [27] has evaluated best effect of nonpharmacological therapies. Evidence of potential grade A treatment recommendation was reported for the effect of multicomponent intervention in delaying institutionalisation and grade B treatment recommendation for improvement in cognition and of activity of daily living. The intervention program of the present study is in accordance with these recommendations.

Several events and mechanisms may explain the similar cognitive performances between participants receiving stimulation therapy and standard care. In our study all groups were engaged in some treatment regimen also the standard care group that received placebo. We suggest that even this group was exposed to more than standard care throughout the study period because of overly enthusiastic co-workers including test technicians who became involved in activities that exceeded their predefined roles. Frequent monitoring and follow-ups do increase the attention given to the patients by family members, caregivers and study staff. This may act as stimulation in itself and generate an expectancy of a beneficial outcome [28]. We know from the experiences of industrial companies that production may increase no matter what changes are introduced to the workers or working conditions, “the Hawthorne effect” [29]. The Hawthorn effect has to our knowledge only been addressed in one single AD study [30]. This effect is not sufficiently described in clinical AD trials [31,32], and it may contribute to controls performing better than expected.

Beside the above-mentioned mechanisms, our study results may have been affected by a national AD campaign launched at the same time. That campaign focus on the beneficial effects of cognitive stimulation. This could have contributed to a diffusion of the therapeutic procedures across municipality borders and diluted the differences between stimulation therapy and standard care.

### Strengths and weaknesses

Our study is population-based with few exclusion criteria. This is different from several other studies which recruited AD individuals from hospitals, memory clinics or nursing homes using restrictive inclusion and wide exclusion criteria that could influence study samples and results. Our study population is ethnically and socially homogenous, and the baseline characteristics did not differ between defined strata. The two-by-two factorial design in our study enables a head to head comparison between stimulation therapy and drug treatment

Participants remained in their own environment during the entire study period. None of the participants used memantin or other ChEI. Although 23% of the participants used anticholinergic drugs for co-morbidities [33], inappropriate drugs were equally distributed between groups and could hardly explain the results.Our participants received structured stimulation therapy on a daily basis and had few dropouts (22%), and the dropouts were equally distributed among subgroups. A possible weakness is that lower MMSE score among dropouts could have influenced mean cognitive deterioration during follow-up. However, repeated measure analyses confirmed the main result of the study.

Another possible weakness is that despite randomisation, participants in the donepezil group had a lower educational level compared to the placebo group. Less education is associated with an increased risk of AD [2] but it is questionable whether this risk factor has an influence on the cognitive trajectory in AD in any way. The non-randomized allocation of the participating municipalities to stimulation therapy or standard care could be considered a weakness. However, a sensitivity analysis done to control for possible clustering of data within the municipalities did not change the results. The ability of the applied tests to detect a change in cognitive performance is questioned in early stage AD [18]. However, a stratified analysis of a subgroup presenting a MMSE score less than 21 points at entry (n = 43) showed no differences, and the results were consistent for all three cognitive tests (data not shown). It is therefore unlikely that a MMSE learning effect has occurred.

Furthermore, the stratified samples in the two-by-two factorial analysis that we did could be prone to type II errors due to relatively small sample sizes - especially the subgroup analyses of participants with MMSE < 21. However, the differences between all groups were consistent for all three tests. Another consequence of the two-by-two factorial design was that no subgroup was left without any intervention. Even the standard care group received either placebo or donepezil. This could have increased the expectancy of a favourable outcome in the control groups and diluted the results [28].

## Conclusion

Participants with recently diagnosed AD receiving stimulation therapy retained cognitive function during the one-year follow-up as did also AD participants receiving standard care. Donepezil therapy had no additional effect on cognition. Our results need to be confirmed in future studies.

## Abbreviations

AD: Alzheimer’s disease; ADAS-cog: Alzheimer’s disease Assessment Scale cognitive (Scale 0―70: increasing disability with increasing score); BI: Barthel Index (Scale 0―20 better function with increasing score); CDT: Clock Drawing Test; ChEI: Cholinesterase inhibitor; DSM-IV-TR: Statistical Manual of Mental Disorders fourth edition; GP: General Practitioner; ICD-10: International classification of diseases 10th Revision; IQ-CODE: Informant Questionnaire-Cognitive Decline in the Elderly; MADRS: Montgomery and Aasberg Depression Rating (Scale 0―60 increasing depression by increasing number); MMSE: Mini-Mental State Examination (Scale 0―30 better function with increasing score); NPI: NeuroPsychiatric Inventory (Scale 0―144 increasing number of psychiatric symptoms by increasing number); NINCDS-ADRDA: National Institute of Neurological Disorders and Stroke-Alzheimer Disease’s and Related Disorders Assosiation; RCT: Randomised clinical trial; SD: Standard Deviation.

## Competing interests

The authors declare that they have no competing interests.

## Authors’ contributions

FA has initiated, coordinated and conducted this study in close co-operation with the scientific advisory board at The University of Tromsø. He has examined and diagnosed patients recruited both in general practice and by postal cognitive screening. He is also responsible for analyzing baseline data and analyzing the main results of the study. TE has been the main supervisor and member of the scientific advisory board, participating in all stages of this study; - planning, lecturing, collecting data, discussing results and writing. BS participated in the planning of the study, supervising implementation and analysis and has revised the manuscript. He is a member of the scientific advisory board. MV is a member of the scientific advisory board and has participated in diagnosing AD participants, and in revising this manuscript. DSH has participated in the data analysis, and has contributed significantly in drafting and writing of the paper. TW has supervised and verified the main statistical analyses and participated in the interpretation of the results. All authors have full access to all the data (including statistical reports and tables) and have approved the final version of the paper.

## Funding

The Northern Norway Regional Health Authority, The National Centre of Rural Health at The University of Tromsø, Health and Rehabilitation, The Directory of Health and Social Welfare in Norway, The County Officer of Nordland and The Municipality of Steigen constituted the funding group. Pfizer delivered donepezil and placebo, but had otherwise no influence on the study design, data collection, analyses and publication.

## Pre-publication history

The pre-publication history for this paper can be accessed here:

http://www.biomedcentral.com/1471-2377/12/59/prepub
